# Aerobic Exercise Ameliorates Myocardial Inflammation, Fibrosis and Apoptosis in High-Fat-Diet Rats by Inhibiting P2X7 Purinergic Receptors

**DOI:** 10.3389/fphys.2019.01286

**Published:** 2019-10-11

**Authors:** Xudong Chen, Haiyan Li, Kangwei Wang, Xiaohe Liang, Weiqi Wang, Xiaokang Hu, Zhouqing Huang, Yonghua Wang

**Affiliations:** ^1^The Key Lab of Cardiovascular Disease of Wenzhou, Department of Cardiology, Wenzhou Medical University, Wenzhou, China; ^2^Department of Cardiology, Ningbo Hangzhou Bay Hospital, Ningbo, China; ^3^Department of Rehabilitation, The First Affiliated Hospital of Wenzhou Medical University, Wenzhou, China; ^4^Department of Cardiology, The First Affiliated Hospital of Wenzhou Medical University, Wenzhou, China; ^5^Department of Physical Education, Wenzhou Medical University, Wenzhou, China

**Keywords:** aerobic exercise, P2X7 purinergic receptors, high-fat-diet, inflammation, fibrosis, apoptosis

## Abstract

**Background:**

High-fat-diet (HFD) is associated with chronic low-grade inflammation. P2X7 purinergic receptors (P2X7R) are key regulators of inflammasome activation. The benefits of exercise are partly attributed to its anti-inflammatory effect, but whether it regulates P2X7R expression to improve remodeling in cardiac myocytes treated by HFD is not completely clarified.

**Methods:**

Three groups of Sprague-Dawley (SD) rats were studied: (1) control group (fed a normal chow diet), (2) HFD group, and (3) HFD+ exercise group. H9c2 myocytes were pretreated with or without A438079 (a P2X7R inhibitor) and then exposed to 200 μM palmitic acid (PA) for 24 h. The levels of mRNA and protein were measured by real-time PCR and Western blot, respectively. Masson staining and hematoxylin-eosin (HE) staining were used to identify remodeling of the heart. The concentration of IL-1β in serum or supernatants were measured by ELISA.

**Results:**

*In vivo*, collagen deposition and the number of disordered cells significantly increased in the hearts of the HFD group compared to the control group. However, exercise markedly reversed these changes in the myocardium, and the same trends were observed in the expression of MMP9, collagen I and TGF-β. Notably, the expression of P2X7R, NLRP3, caspase-1 in the hearts, and serum IL-1β level were also greatly upregulated in the heart of the HFD diet rats, and all these changes were ameliorated in the HFD + EX group. As expected, exercise also reduced the number of TUNEL-positive cells, which was consistent with the caspase-3, Bax, and Bcl-2 results. Moreover, exercise reduced body weight and blood lipid concentrations in the HFD diet rats. *In vitro*, we observed that the hallmark of fibrosis, inflammation and apoptosis in H9c2 myocytes enhanced by PA, and the P2X7R inhibitor treatment significantly reduced the expression of the NLRP3, caspase-1, suppressed the secretion of IL-1β of H9c2 cells, inhibited collagen I, TGF-β, MMP9, Bax, caspase-3 levels and increased the expression of Bcl-2, compared with the PA group. In addition, a decrease of the number of TUNEL-positive cells used by A438079 further support that cardiomyocytes apoptosis could be inhibited.

**Conclusion:**

Aerobic exercise reversed the cardiac remodeling via the reduction of inflammation, fibrosis and apoptosis in HFD rats, at least in part through inhibiting P2X7R expression in cardiomyocytes.

## Introduction

Long-term high-fat-diet (HFD) can lead to weight gain and a series of adverse reactions, including obesity, hyperlipidemia and hyperglycemia (Carlin et al., 2016). Additionally, obesity is closely related to type 2 diabetes, hypertension, heart failure, and coronary heart disease (Wilson et al., 2002; Noyan-Ashraf et al., 2013; Riehle and Abel, 2016). All of these can cause cardiac injury. Myocardial fibrosis and remodeling occur under various adverse stimuli, accompanied by systolic and diastolic dysfunction, arrhythmia and adverse outcomes (Frangogiannis, 2019). Alarmingly, obesity alters gene expression in the heart of offspring (Raipuria et al., 2015). Emerging evidence has demonstrated that obesity is associated with chronic low-grade inflammation because excessive fat causes abnormal function and increases the level of systemic proinflammatory factors (Deng et al., 2016; Shan et al., 2017), that are associated with the progression of pathophysiological processes such as myocardial dysfunction. Consequently, an effective method to control obesity and reduce cardiac injury would be highly desirable.

P2X7 purinergic receptors (P2X7R), ligand gated ion-channels, are the key regulator of interleukin-1β (IL-1β) cleavage, which are important contributors to control the progression of obesity (Stienstra et al., 2012). After the binding of extracellular adenosine triphosphate (ATP), the ion channel opens, allowing calcium influx and potassium efflux to induce inflammasome activation and IL-1β release (Stachon et al., 2017), which leads to cell death (Di Virgilio et al., 2017). P2X7R contributes to chronic inflammation in different disease models such as inflammatory bowel diseases, acute pancreatitis and atherosclerosis (Hoque et al., 2011; Gulbransen et al., 2012; Stachon et al., 2017). In addition, it has been reported that P2X7R is involved in renal fibrosis, and knockout of P2X7R can delay the process of it (Solini et al., 2013). However, the potential role of P2X7R in the heart of HFD rats remains unclear.

Numerous methods were used to treat obesity including lifestyle changes (dietary intervention and moderate exercise), psychological and behavioral interventions, pharmacologic treatment and surgical intervention (Dyson, 2010). Exercise is a simple and effective approach for treatment of obesity. Regular physical activity has many beneficial effects. For example, it ameliorated diabetic cardiomyopathy by improving mitochondrial function and cardiac contractility (Ko et al., 2017), prevented metabolic syndrome, hypertension, and obesity (Cho et al., 2017) and also attenuated aortic endothelial oxidative damage induced by hyperhomocysteinemia (Chan et al., 2018). The benefits of exercise are partly attributed to its anti-inflammatory effects. In nervous system, exercise reduced prefrontal cortex inflammation by suppressing C-X-C motif ligand 10 (CXCL10) and C-C motif ligand 2 (CCL2) in HFD mice (Carlin et al., 2016), as well as substantia nigra dopaminergic neurons inflammation by activating brain-derived neurotrophic factor (BDNF) signaling pathway in lipopolysaccharide (LPS) treatment mice (Wu et al., 2011). In immune system, exercise reduced tumor necrosis factor (TNF) production in splenic by activating the subdiaphragmatic vagus nerve (Shimojo et al., 2019). Exercise also inhibited TNF expression in rheumatic diseases by stimulating muscle production of interleukin-6 (IL-6) and by promoting the production of interleukin-1 receptor antagonist (IL-1Ra), and interleukin-10 (IL-10) (Benatti and Pedersen, 2015). In circulatory system, exercise mitigated inflammation in cardiac tissue and improved left ventricular function by increasing IL-10 in HFD mice (Kesherwani et al., 2015). Taken all, regular exercise may be an effective treatment for some diseases, but its mechanism need to further clarify. In this study, we investigated the effect of aerobic exercise on P2X7R expression and regulating the cardiac remodeling of HFD rats, explored whether its underlying mechanism of exercise is involved in the regulation of P2X7R.

## Materials and Methods

### Reagents

Dulbecco’s modified Eagle medium (DMEM), fetal bovine serum (FBS), and penicillin/streptomycin (pen/strep, 10,000 U/ml and 10,000 ug/ml, respectively) were purchased from the GIBCO Company (Shanghai, China). Palmitic acid (PA) was obtained from Sigma-Aldrich (Shanghai, China). A438079 (S7705) was obtained from Selleck (Shanghai, China). P2X7R (#APR-004) antibody was obtained from Alomone Laboratories (Jerusalem, Israel). NOD-like receptor protein 3 (NLRP3) (NBP2-12446) antibody was obtained from Novus Biologicals (Littleton, CO., United States). Collagen 1 (ab34710), matrix metalloproteinases (MMP9) (ab38898) and Bcl-2 (ab196495) antibodies were obtained from Abcam (Cambridge, United Kingdom). GAPDH (#5174), TGF-β (#3711), Caspase-3 (#9662), and Bax (#14796) antibodies were bought from Cell Signaling Technology (Danvers, MA, United States). Caspase-1 (sc-56036) was bought from Santa Cruz Biotechnology (Santa Cruz, CA, United States). Cell Counting Kit-8 (C0038), goat anti-rabbit secondary antibodies (A0208), and goat anti-mouse secondary antibodies (A0216) used in the Western blot and One Step TUNEL Apoptosis Assay Kit were obtained from Beyotime (Shanghai, China). Hematoxylin-eosin (HE) Staining Kit and Masson’s Trichrome Stain Kit were obtained from Solarbio (Beijing, China). Triglyceride (OSR61118), total cholesterol (OSR6216) was obtained from Beckman Coulter (Jiangsu, China), low-density lipoprotein (LDL) (483776) and high-density lipoprotein (HDL) (487101) was obtained from the Sekisui Medical Technology (Beijing, China).

### Treatment and Culture of Cardiomyocytes

H9c2 myocytes was purchased from American Type Culture Collection (Rockville, MD, United States). H9c2 myocytes were maintained in DMEM containing 4.5 g/L of glucose and supplemented with 10% (v/v) FBS and 1% penicillin/streptomycin at 37°C in a humidified 5% CO_2_ incubator. The cardiomyocytes were pretreated with A438079 (a selective P2X7R inhibitor) for 12 h before addition of PA, after that, the cardiomyocytes were stimulated with PA (200 μM) for 24 h.

### Animals

This study was carried out in accordance with the principles of the Guide for the Care and Use of Laboratory Animals (National Research Council, United States). The protocol was approved by the Institutional Animal Care and Use Committee, Wenzhou Medical University (wydw2016-0266). Six-week-old male Sprague-Dawley (SD) rats (*n* = 24) were purchased from the Animal Center of Wenzhou Medical University and housed in specific pathogen-free (SPF) conditions. The animals were kept under a 12-h/12-h light–dark cycle and were allowed free access to food and water.

### Experimental Exercise Protocol

After 1 week of acclimatization, the rats were fed either a control diet (*n* = 8) or a HFD (MD12032; Medicine, Jiangsu, China) (*n* = 16). Twelve weeks later, HFD rats were randomly divided into two groups: sedentary rats fed the HFD (*n* = 8) and regular aerobic exercise trained rats fed the high-fat diet (HFD + EX) (*n* = 8). The sedentary rats fed a standard diet served as the control (CON) (*n* = 8). The rats in the exercise groups were trained on a motor treadmill at the speed of 5 m/min for 60 min on the first day. The running speed was increased 1 m/min each day until the speed reached 10 m/min at the end of the training protocol (Fernando et al., 1993). These rats were trained 7 days/week for 12 weeks. All training sessions took place during the afternoon (2:00–5:00 p.m.). After 12 weeks of the treadmill exercise, the rats were anesthetized with an intraperitoneal injection of pentobarbital sodium (50 mg/kg) after 12 h of starvation. The blood samples were collected from the inferior vena cava into EDTA tubes. The plasma was immediately separated by centrifugation at 3000 rpm for 10 min and kept frozen at −80°C until chemical assay analysis (Supplementary Figure S1).

### Real-Time PCR Analysis

The hearts from the SD rats were used to prepare total RNA using TRIzol Reagent according to the manufacturer’s protocol (Invitrogen Life Technologies). One microgram of total RNA from each sample was used to generate cDNAs using the RevertAidTM First Strand cDNA Synthesis Kit (#K1622; Thermo). The resultant cDNA was amplified by SYBR (#RR037A; Takara). The PCR was directly monitored by the CFX96 Touch^TM^ Real-Time PCR detection system. All results were normalized against GAPDH (B661204; Sangon Biotech, Shanghai, China).

The real-time polymerase chain reaction used the following primers:

P2X7R: Forward primer 5′-CGGGCCACAACTATACT ACGA-3′
Reverse primer 5′-CCTGAACTGCCACCTCT GTAA-3′
NLRP3: Forward primer 5′-GAGCTGGACCTCAGTG ACAATGC-3′
Reverse primer 5′-ACCAATGCGAGATCCTG ACAACAC-3′
Caspase-1: Forward primer 5′-AGGAGGGAATATGTGG G-3′
Reverse primer 5′-AACCTTGGGCTTGTCT T-3′
MMP9: Forward primer 5′-AAGGATGGTCTACTGG CAC-3′
Reverse primer 5′-AGAGATTCTCACTGGG GC-3′
Caspase-3:  Forward primer 5′-AACGGACCTGTGGACCT GAA-3′
Reverse primer 5′-TCAATACCGCAGTCCAG CTCT-3′
Bcl-2: Forward primer 5′-CCGGGAGAACAGGG TATGATAA-3′
Reverse primer 5′-CCCACTCGTAGCCCCT CTG-3′
Bax:Forward primer 5′-GATCAGCTCGGGCAC TTTA-3′
Reverse primer 5′-TGTTTGCTGATGGCAA CTTC-3′
Collagen I: Forward primer 5′-TGACGCATGGCCAAGA AGAC-3′
Reverse primer 5′-CCGTGCCATTGTGGCAG ATA-3′
TGF-β: Forward primer 5′-GACTCTCCACCTGCAA GACC-3′
Reverse primer 5′-GGACTGGCGAGCCTTA GTTT-3′
GAPDH: Forward primer 5′-GACATGCCGCCTGGA GAAAC-3′
Reverse primer 5′-AGCCCAGGATGCCCTT TAGT-3′

### Western Blot Analysis

The heart tissue samples (50–100 mg) and cardiomyocytes samples were lysed and centrifuged at 12,000 × *g* for 15 min, and then the supernatants were collected. The protein samples were separated by SDS-PAGE gel and transferred to a PVDF membrane (MERCK, Germany). The membrane was blocked with a 5% fat-free milk solution for 1 h at room temperature and subsequently incubated overnight at 4°C with the primary antibodies. After washing three times, immunoreactive bands were incubated with horseradish peroxidase (HRP)-conjugated secondary antibody. The proteins were detected via the ECL procedure (Bio-Rad, United States).

### TUNEL Staining

The terminal deoxynucleotidyl transferase-mediated DUTP nick end labeling (TUNEL) assay was performed using the One Step TUNEL Apoptosis Assay Kit (Beyotime, Shanghai, China) according to the manufacturer’s instructions. TUNEL positive cells were imaged under a fluorescence microscope (Nikon, Japan).

### Masson and HE Staining

Fresh tissue was fixed in 4% paraformaldehyde and embedded in paraffin. Five-micron sections were obtained, deparaffinized, and rehydrated as previously described. After rehydration, the sections were stained with HE. To evaluate the level of collagen deposition and fibrosis, paraffin sections were stained using a Masson’s Trichrome Stain Kit. The stained sections were then viewed under the Nikon microscope (Nikon, Japan).

### Assessment of Cell Viability With CCK-8

Cell viability was assessed with the CCK-8 assay according to the manufacturer’s instruction. The kit contains a water-soluble tetrazolium salt (WST-8), a substrate of the mitochondrial succinate dehydrogenases that convert WST-8 into formazan (orange-colored). The activities of these enzymes are proportional to the number of living cells. H9c2 cells were seeded into 96-well plates at a concentration of 5000 cells per well and exposed to various culture conditions, including control, PA or A438079. At the end of each treatment, the culture medium was replaced with 100 μl of CCK-8 solution (containing 90 μl of serum-free DMEM with 10 μl of CCK-8 reagent). The absorbance at 450 nm was measured in each well by visualization of color intensity development.

### ELISA

After various treatments, the concentration of IL-1β in serum or in supernatants were measured by ELISA kits (R&D Systems). Assays were performed according to the protocols included with the kit. Absorbance at 450 nm was measured in each well by visualization of color intensity development.

### Statistical Analysis

All values were presented as the mean ± SEM and statistical analyses were performed using GraphPad Prism 7 (GraphPad, San Diego, CA, United States). We tested the normality of data distribution using Shapiro-Wilks test. We tested the equal variance using Levene’s test. A one-way ANOVA followed by a multiple comparisons test with a Tukey’s correction was employed to analyze the differences within the multiple groups. A *p*-value <0.05 was considered significant.

## Results

### Changes in Body Weight and Blood Lipid Levels

**FIGURE 1:**
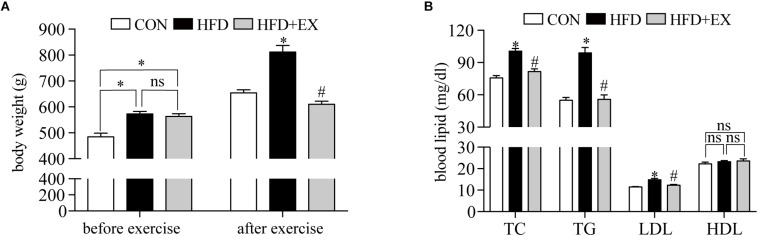
Changes in body weight and blood lipid levels. **(A)** Body weight was measured before and after 12 weeks of the treadmill exercise. **(B)** Blood lipid levels after 12 weeks of the treadmill exercise. TG, triglyceride; TC, total cholesterol; LDL, low-density lipoprotein; HDL, high-density lipoprotein. ^∗^*p* < 0.05 versus the CON group, ^#^*p* < 0.05 versus the HFD group.

We monitor the change of body weight and blood lipid levels before and after exercise. Before starting the treadmill exercise, the body weight of rats from CON, HFD, and HFD + EX group were 484.1 g ± 14.2, 572.0 g ± 10.1, and 561.8 g ± 10.9, respectively, which indicated the rats in HFD and HFD + EX groups were heavier than the CON group (*p* < 0.001), but no significant difference between the HFD and HFD + EX groups was observed (*p* = 0.82) (Figure 1A). At the end of the treadmill exercise period, the body weight of rats from CON, HFD, and HFD + EX group were 654.4 g ± 11.8, 811.3 g ± 24.89, and 610.0 g ± 12.1, respectively, which showed the HFD group weighed more than the CON and HFD + EX groups (*p* < 0.0001), and the rats from HFD + EX groups weighed less than CON group, but no significant difference (*p* = 0.19) (Figure 1A). Figure 1B depicts the effects of exercise on the blood lipid levels. The TG of rats from CON, HFD, and HFD + EX group were 55.1 ± 2.6, 98.9 ± 5.1, and 55.9 ± 4.1, respectively. The TC of rats from CON, HFD, and HFD + EX group were 75.8 ± 2.1, 100.6 ± 2.5, 81.5 ± 2.6, respectively. The LDL of rats from CON, HFD, and HFD + EX group were 11.5 ± 0.2, 14.84 ± 0.6, and 12.4 ± 0.4, respectively. HFD increased blood lipid levels compared with CON group (*p* < 0.05) and exercise reduced blood lipid levels compared with HFD group (*p* < 0.05). No statistically significant differences were observed in HDL levels between CON and HFD (*p* > 0.05). *P-*value between HFD and HFD + EX also >0.05 (Figure 1B).

### Regular Aerobic Exercise Decreased Myocardial Inflammation in HFD Rats

**FIGURE 2:**
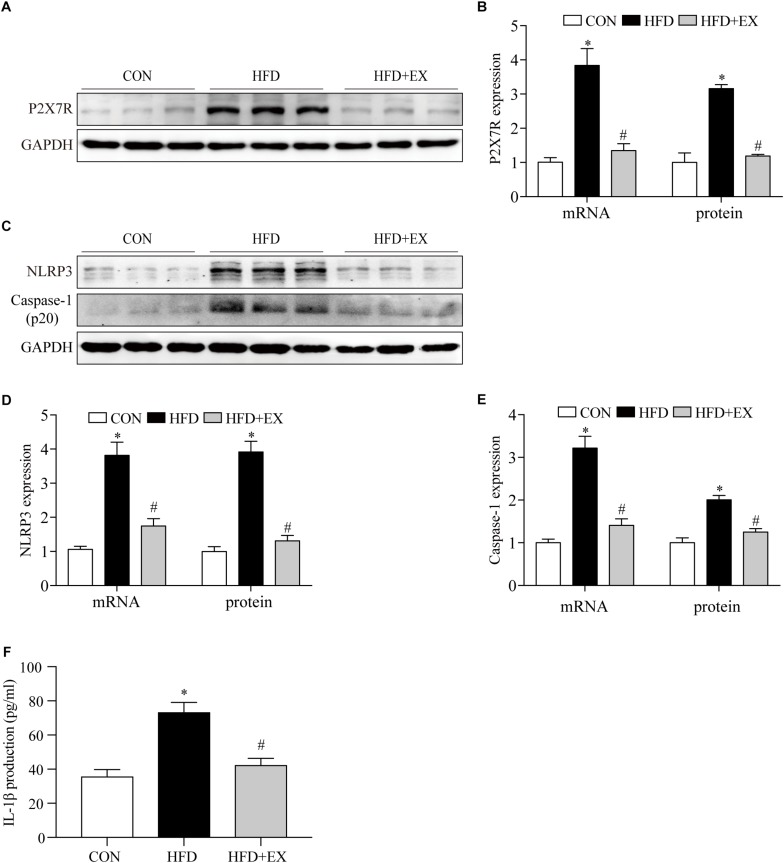
Regular aerobic exercise decreased myocardial inflammation in HFD rats. **(A)** Representative Western blot analysis of P2X7R. **(B)** The mRNA levels and the protein semiquantification are shown for P2X7R. **(C)** Representative Western blot analysis of NLRP3 and caspase-1. **(D)** The mRNA levels and the protein semiquantification are shown for NLRP3. **(E)** The mRNA levels and the protein semiquantification are shown for caspase-1. **(F)** Concentrations of IL-1β in the serum were detected by ELISA. ^∗^*p* < 0.05 versus the CON group, ^#^*p* < 0.05 versus the HFD group.

Exercise has been reported to have anti-inflammatory effects. We examined the expression of P2X7R and downstream proteins in cardiac tissue induced by HFD rats. As seen in Figures 2A,B at the end of the treadmill exercise period, the P2X7R proteins expression of rats’ heart from CON, HFD, and HFD + EX group were 1-fold, 3.1-fold, and 1.2-fold, respectively. The P2X7R mRNA expression of rats from CON, HFD, and HFD + EX group were 1-fold, 3.8-fold, and 1.3-fold, respectively. Under the action of P2X7R, NLRP3 inflammasome was activated, and then cleaved pro-caspase-1 to form caspase-1, which triggered a series of inflammation (Martinez-Garcia et al., 2019). The NLRP3 proteins expression of rats from CON, HFD, and HFD + EX group were 1-fold, 3.9-fold, and 1.3-fold, respectively. The NLRP3 mRNA expression of rats from CON, HFD, and HFD + EX group were 1-fold, 3.8-fold, and 1.7-fold, respectively. The caspase-1 proteins expression of rats from CON, HFD, and HFD + EX group were 1-fold, 2.0-fold, and 1.2-fold, respectively. The caspase-1 mRNA expression of rats from CON, HFD, and HFD + EX group were 1-fold, 3.2-fold, and 1.4-fold, respectively (Figures 2C–E). Briefly, compared with CON group, the expression of NLRP3 and caspase-1 increased in HFD group (*p* < 0.05). Exercise decreased the expression of NLRP3 and caspase-1 (*p* < 0.05). The serum levels of IL-1β in HFD-fed rats from CON, HFD, and HFD + EX group were 35.8 pg/ml ± 4.0, 73.3 pg/ml ± 5.7, and 42.4 pg/ml ± 3.9, respectively (*p* < 0.0001 between CON and HFD group, *p* < 0.001 between HFD and HFD + EX group) (Figure 2F). These data indicate that aerobic exercise decreased myocardial inflammation in HFD rats.

### Regular Aerobic Exercise Decreased Myocardial Remodeling in HFD Rats

**FIGURE 3:**
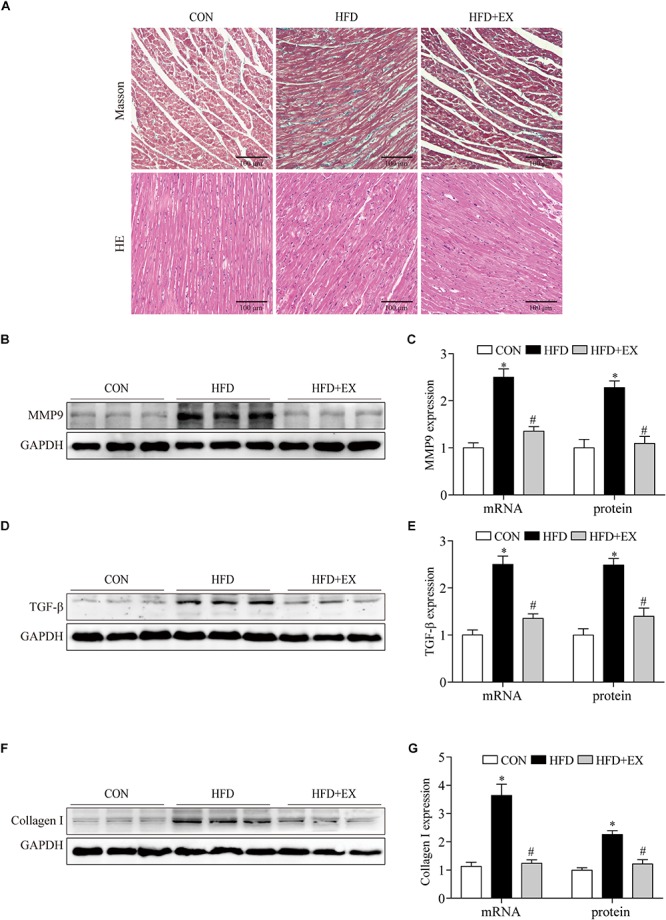
Regular aerobic exercise decreased myocardial remodeling in HFD rats. **(A)** Representative images of Masson stained and HE stained rat left ventricles. **(B)** Representative Western blot analysis of MMP9. **(C)** The mRNA levels and the protein semiquantification are shown for MMP9. **(D)** Representative Western blot analysis of TGF-β. **(E)** The mRNA levels and the protein semiquantification are shown for TGF-β. **(F)** Representative Western blot analysis of collagen I. **(G)** The mRNA levels and the protein semiquantification are shown for collagen I. ^∗^*p* < 0.05 versus the CON group, ^#^*p* < 0.05 versus the HFD group.

To investigate the effect of exercising training on cardiac remodeling of HFD rats, we examined the markers of cardiac fibrosis reflected by protein contents and mRNA of TGF-β, collagen I and MMP9 levels. We observed the collagen deposition in the myocardium by Masson staining and morphological changes by HE staining. We found that both of collagen deposition and the number of disordered cells markedly increased in cardiac tissue of the HFD group, in contrast, regular aerobic exercise reversed these changes (Figure 3A). As seen in Figures 3B–G, at the end of the treadmill exercise, the MMP9 proteins expression of rats’ heart from CON, HFD, and HFD + EX group were 1-fold, 2.3-fold, and 1.1-fold, respectively. The MMP9 mRNA expression of rats from CON, HFD, and HFD + EX group were 1-fold, 2.5-fold, and 1.3-fold, respectively. The TGF-β proteins expression of rats from CON, HFD, and HFD + EX group were 1-fold, 2.5-fold, and 1.4-fold, respectively. The TGF-β mRNA expression of rats from CON, HFD, and HFD + EX group were 1-fold, 2.5-fold, and 1.3-fold, respectively. The collagen I proteins expression of rats from CON, HFD, and HFD + EX group were 1-fold, 2.2-fold, and 1.2-fold, respectively. The collagen I mRNA expression of rats from CON, HFD, and HFD + EX group were 1-fold, 3.6-fold, and 1.2-fold, respectively. Briefly, compared with CON group, HFD increased the expression of MMP9, TGF-β and collagen I (*p* < 0.05), and exercise decreased the expression of these proteins (*p* < 0.05). Taken together, these results indicated that HFD induced fibrosis is attenuated by exercise training.

### Regular Aerobic Exercise Inhibited Myocardial Apoptosis in HFD Rats

**FIGURE 4:**
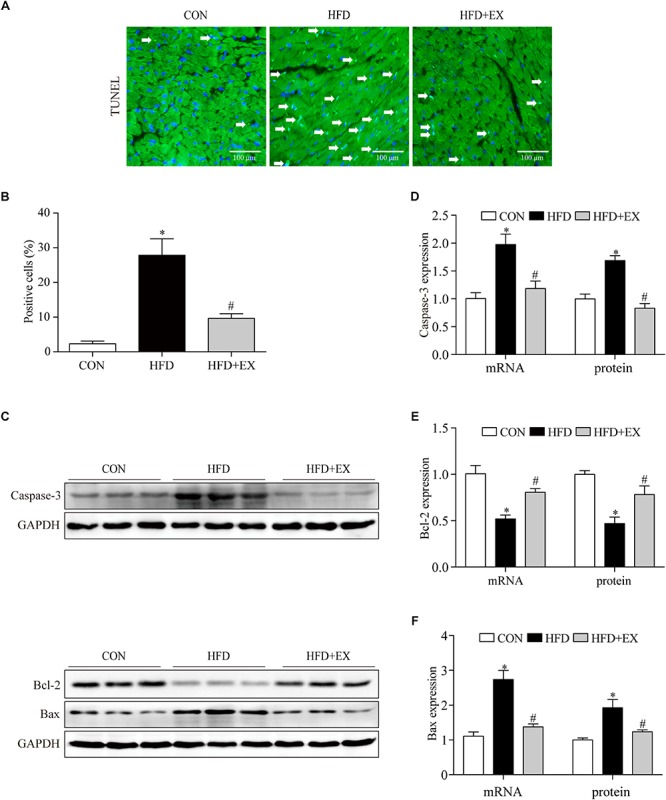
Regular aerobic exercise inhibited myocardial apoptosis in HFD rats. **(A)** Representative TUNEL stained images of rat left ventricles. **(B)** The percentage of TUNEL-positive cells are shown. **(C)** Representative Western blot analysis of caspase-3, Bcl-2, and Bax. **(D)** The mRNA levels and the protein semiquantification are shown for caspase-3. **(E)** The mRNA levels and the protein semiquantification are shown for Bcl-2. **(F)** The mRNA levels and the protein semiquantification are shown for Bax. ^∗^*p* < 0.05 versus the CON group, ^#^*p* < 0.05 versus the HFD group.

To investigate the effect of exercise on the myocardial apoptosis, we tested photomicrographs of TUNEL-positive cells in the myocardium. Photomicrographs of TUNEL-positive cells in the myocardium are presented in Figures 4A,B. In the CON group, the TUNEL-positive cells were barely detected, and the numbers of TUNEL-positive cells in the HFD group were significantly higher than the CON group (2% in CON group vs. 35% in HFD group, *p* < 0.001). In contrast, regular aerobic exercise reduced the increased positive cells to 6% compared to the HFD group (*p* < 0.001). Furthermore, HFD significantly upregulated the expression of caspase-3 and Bax, while exercise reversed these changes. The caspase-3 proteins expression of rats from CON, HFD, and HFD + EX group were 1-fold, 1.7-fold, and 0.8-fold, respectively. The caspase-3 mRNA expression of rats from CON, HFD, and HFD + EX group were 1-fold, 1.9-fold, and 1.2-fold, respectively. The Bax proteins expression of rats from CON, HFD, and HFD + EX group were 1-fold, 1.9-fold, and 1.2-fold, respectively. The Bax mRNA expression of rats from CON, HFD, and HFD + EX group were 1-fold, 2.7-fold, and 1.4-fold, respectively. Moreover, HFD downregulated the expression of Bcl-2, while exercise upregulated its expression. The Bcl-2 proteins expression of rats from CON, HFD, and HFD + EX group were 1-fold, 0.4-fold, and 0.8-fold, respectively. The Bcl-2 mRNA expression of rats from CON, HFD, and HFD + EX group were 1-fold, 0.5-fold, and 0.8-fold, respectively. Briefly, compared with CON group, HFD increased the expression of caspase-3, Bax (*p* < 0.05) and decreased the expression of Bcl-2. Exercise reversed the expression of these proteins (*p* < 0.05) (Figures 4C–F). Taken together, these results indicated that HFD induced apoptosis is attenuated by exercise training.

### P2X7R Inhibitor Suppressed Cardiomyocyte Inflammation, Fibrosis and Apoptosis Induced by PA

Since HFD upregulated the expression of P2X7R and exercise greatly decreased the levels of P2X7R in HFD rats, we hypothesized that P2X7R may play a role in the pathophysiological processes induced by HFD.

**FIGURE 5:**
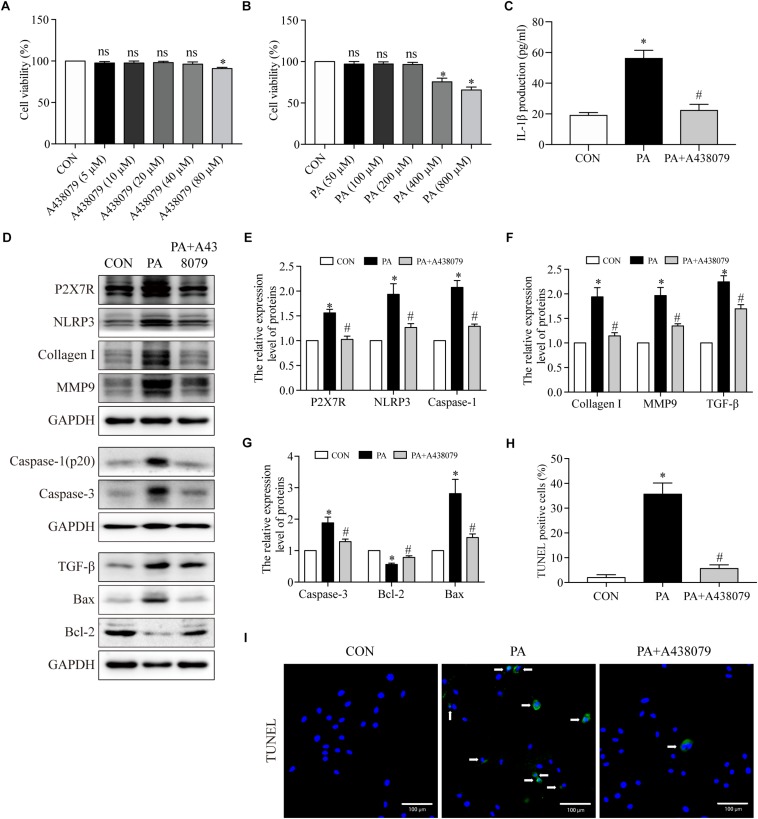
The P2X7R inhibitor suppressed PA-induced inflammation, fibrosis and apoptosis in cardiomyocytes. **(A)** Cell viability was assessed by the CCK8 assay. H9c2 myocytes were incubated in a medium with A438079 at 0–80 μM for 12 h, and cells without A438079 treatment served as the control. **(B)** H9c2 myocytes were incubated in a medium with PA at 0–800 μM for 24 h, and cells without PA treatment served as the control. **(C)** Concentrations of IL-1β in cell culture supernatants were detected by ELISA. **(D)** Representative Western blot analysis of P2X7R, NLRP3 inflammasome, collagen I, MMP9, TGF-β, caspase-1, caspase-3, Bax, and Bcl-2. **(E)** The protein semiquantification is shown for P2X7R, NLRP3 and caspase-1. **(F)** The protein semiquantification is shown for collagen I, MMP9 and TGF-β. **(G)** The protein semiquantification is shown for caspase-3, Bax, and Bcl-2. **(H)** The percentages of TUNEL-positive cells are shown. **(I)** Representative images of TUNEL stained H9c2 myocytes. CON, H9c2 myocytes without any treatment; PA, H9c2 myocytes treated with 200 μM PA for 24 h; PA + A438079, H9c2 myocytes pretreated with A438079 for 12 h, and then treated with 200 μM PA for 24 h. ^∗^*p* < 0.05 versus the CON group, ^#^*p* < 0.05 versus the HFD group.

To further investigated the function of P2X7R on cardiomyocytes underlying the HFD environment, we used PA to simulate lipotoxicity in H9c2 myocytes (Roychowdhury et al., 2016). The effect of A438079 (P2X7R inhibitor) on the cell viability of H9c2 cells were measured by CCK8 assay, with the concentration ranging from 5 to 80 μM for 12 h. No significant differences in cell viability were observed in response to as much as 40 μM A438079 for 12 h (Figure 5A). Therefore, we chose a concentration of 40 μM A438079 to inhibit the expression of P2X7R. The same method was used to measure the effect of PA on cell viability. No significant differences in cell viability were observed in response to up to 200 μM of PA for 24 h (Figure 5B). We therefore used 200 μM PA for the subsequent study.

To confirm the role of P2X7R in inflammation, fibrosis and apoptosis in cardiomyocytes induced by PA, we first observed that the PA-induced group had higher level of IL-1β in the supernatant of H9c2 compared to CON group (56.4 pg/ml ± 5.1 vs. 19.3 pg/ml ± 1.6, *p* < 0.001) (Figure 5C), which was reduced to 22.5 pg/ml ± 3.7 when the cells were treated with A438079 (*p* < 0.01). In addition, as depicted in Figures 5D–F, compared with control group, the P2X7R, NLRP3, caspase-1, collagen I, MMP9, and TGF-β expression were greatly upregulated in H9c2 myocytes after stimulation with PA. The expression of these proteins was significantly inhibited by A438079. The P2X7R proteins expression from CON, PA, and PA + A438079 group were 1-fold, 1.5-fold, and 1-fold, respectively. The NLRP3 proteins expression from CON, PA, and PA + A438079 group were 1-fold, 1.9-fold, and 1.3-fold, respectively. The caspase-1 proteins expression from CON, PA, and PA + A438079 group were 1-fold, 2.0-fold, and 1.3-fold, respectively. The collagen I proteins expression from CON, PA, and PA + A438079 group were 1-fold, 1.9-fold, and 1.1-fold, respectively. The MMP9 proteins expression from CON, PA, and PA + A438079 group were 1-fold, 1.9-fold, and 1.3-fold, respectively. The TGF-β proteins expression from CON, PA, and PA + A438079 group were 1-fold, 2.2-fold, and 1.7-fold, respectively. Briefly, compared with CON group, PA increased the expression of P2X7R, NLRP3, caspase-1, collagen I, MMP9, TGF-β (*p* < 0.05), and P2X7R inhibitor decreased the expression of these proteins (*p* < 0.05) (Figures 5D–F). These findings reflected that P2X7R is involved in the activation of fibrosis and inflammation upon PA stimulation.

Additionally, A438079 treatment diminished the number of TUNEL-positive cells after PA stimulation. The percentage of TUNEL positive cells from CON, PA, and PA + A438079 group were 2, 36, and 6% respectively (*p* < 0.001 between CON and PA group, *p* < 0.001 between PA and PA + A438079 group) (Figures 5H,I). Concomitantly, A438079 downregulated the expression of caspase-3, Bax (pro-apoptotic protein), and upregulated the expression of Bcl-2 (anti-apoptotic protein) in H9c2 cells stimulated by PA (Figures 5D,G). The caspase-3 proteins expression from CON, PA, and PA + A438079 group were 1-fold, 1.9-fold, and 1.3-fold, respectively. The Bax proteins expression from CON, PA, and PA + A438079 group were 1-fold, 2.8-fold, and 1.4-fold, respectively. The Bcl-2 proteins expression from CON, PA, and PA + A438079 group were 1-fold, 0.6-fold, and 0.8-fold, respectively. Briefly, compared with CON group, PA increased the expression of caspase-3, Bax (*p* < 0.05), and decreased the expression of Bcl-2 (*p* < 0.05), P2X7R inhibitor reversed these changes (*p* < 0.05). These data indicate that P2X7R is involved in the activation of apoptosis upon PA stimulation.

## Discussion

In the present study, we found that the expression of P2X7R, NLRP3, caspase-1 and serum IL-1β level was greatly increased, companied by enhancing fibrosis and apoptosis in the hearts induced HFD, and exercise training markedly reversed these changes. Moreover, *in vitro*, the hallmark of fibrosis, inflammation and apoptosis in H9c2 myocytes enhanced by PA, and P2X7R level also upregulated. In addition, the P2X7R inhibitor treatment significantly reduced the expression of the NLRP3, caspase-1, suppressed the secretion of IL-1β, inhibited apoptosis and fibrosis. These findings suggested that aerobic exercise reversed the cardiac remodeling via the reduction of inflammation, at least in part through inhibiting P2X7R expression in cardiomyocytes.

Previous reports have demonstrated that obesity is associated with chronic inflammation (Dake and Oltman, 2015), which includes increasing circulating inflammatory cytokines (Saltiel and Olefsky, 2017) and leukocyte recruitment to inflammatory tissues (Lee et al., 2016). These processes initiate cardiomyocyte fibrosis and apoptosis that ultimately progress to cardiac dysfunction (Carvalheira et al., 2013). As we known, there has established a link between chronic inflammation and obesity-associated complications (Lumeng and Saltiel, 2011; Wu and Ballantyne, 2017). Wang et al. (2017) showed that the HFD-associated myocardial inflammatory injury was likely attributed to both direct and indirect effects of PA on cardiac cells, which including, inflammatory responses, companied by hypertrophy, fibrogenic changes and apoptosis of cardiomyocytes. And anti-inflammatory treatment with L6H9 effectively reduced the PA-increased fibrogenic protein levels and apoptosis. These results provide support explain, at least in part, that chronic inflammation is closely related to fibrosis and apoptosis.

IL-1β is a key cytokine that regulates the pathogenesis of many inflammatory diseases (Dinarello, 2011), for example, it is closely related to obesity-induced inflammation (Stienstra et al., 2012). Our study showed that HFD stimulated the release of IL-1β into the blood and promotes the activation of systemic inflammation. It was indicated that there are several key processes before IL-1β secretion, the activation of NF-κB increases synthesis of NLRP3 and pro-IL-1β (Stienstra et al., 2012), and the NLRP3 inflammasome, plays an important role in IL-1β cleavage (Afonina et al., 2017). Meanwhile P2X7R, as the key regulator of scission of IL-1β, is involved in the activation of NLRP3 inflammasome, contributing to chronic inflammatory disorders (Stachon et al., 2017). So, an axis exists among P2X7R, NLRP3, and IL-1β plays an important role in different pathophysiological process. Peng K et al. indicated that P2X7R regulates NLRP3 inflammasome activation and promotes the progression of atherosclerosis (Peng et al., 2015). And P2X7R knockout reduced inflammatory levels of glomeruli in glomerulonephritis mice (Di Virgilio et al., 2017), attenuated pulmonary inflammation in smoke-induced lung inflammation and emphysema (Taylor et al., 2009). These data means that P2X7R may be as an important target to regulate inflammatory response for disease intervention. In this report, high expression of P2X7R, caspase-1 and NLRP3 in cardiac tissues was induced by HFD, and this finding is consistent with the observation in H9c2 cardiomyocytes stimulated with PA. This suggests that P2X7R expression likely reflect proinflammatory injury in HFD-induced heart.

In addition, previous studies have also shown that P2X7R participants in cells apoptosis such as dendritic cells (Lucattelli et al., 2011) and retinal ganglion cells (Coutinho-Silva et al., 1999). Briefly, Zhang et al. (2018a) reported (Xue et al., 2016) that miR-187 inhibits retinal cell apoptosis by negative regulating P2X7R, and silencing P2X7R reduced oxidative stress-induce retinal ganglion cells apoptosis via p38 MAPK pathway (Zhang et al., 2018a). Our findings are in line with these studies showing that use of P2X7R inhibitor attenuated apoptosis induced by PA. And it’s reported that IL-1β is an important proinflammatory mediator (Zhang et al., 2018b), its antagonist depress cardiomyocyte apoptosis induced by myocardial infarction (Grebe et al., 2018). In diabetic cardiomyopathy, IL-1β promotes cardiomyocyte apoptosis by inducing endoplasmic reticulum stress (Abbate et al., 2008). These data indicated that the intervention of IL-1β level is effective for some disease. Considering the relationship of P2X7R regulating IL-1β level, which subsequently adjust cell apoptosis, there is a closely link between inflammation and apoptosis. Our studies have provided experimental evidence that P2X7R inhibitor reduced IL-1β secretion from cardiomyocytes into the supernatant. At the same time, all of the activated caspase-1, the executioner of IL-1β cleavage and NLRP3 expression were decreased. Based on the strong relationship between P2X7R and inflammatory response, and the reduction of IL-1β level effectively cardiomyocyte apoptosis, we indicated that P2X7R regulated apoptosis by depressing the production of IL-1β.

Also, knocking out P2X7R inhibits pulmonary fibrosis (Liu et al., 2015), and P2X7R levels contributes to the release of MMP9, an important mediator of myocardial remodeling (Riteau et al., 2010). And our previous research showed that the inhibition of P2X7R downregulated both EMMPRIN and MMP9 expression (Gu and Wiley, 2006; Yabluchanskiy et al., 2013), and reduced NLRP3 inflammasome activation in PMA-induced macrophages (Lin et al., 2018). Intriguingly, inflammatory factors such as TNF-α induce MMP9 activity (Kong et al., 2016), which promote the activation of TGF-β that subsequently enhances the synthesis of collagen I and deregulates collagen turnover, resulting in the development of fibrosis in the heart. As expected, P2X7R inhibition by A438079 reduced MMP9 expression in PA-stimulated H9c2 cells, which support that a decrease in the level of TGF-β and collagen I may at least partly ascribe to the reduction of MMP9. Moreover, we also found silencing NLRP3 significantly reduced the expression of EMMPRIN and MMP9 in monocytes (no published data). Given that the inhibition of P2X7R contributed to the NLRP3 reduction, followed by decreasing EMMPRIN and MMP9 level in monocytes, we inferred that the same link among P2X7R, NLRP3 inflammasome and MMP9 existed, and then regulated the fibrosis in cardiomyocytes induced by PA. Taken all these results, the reduction of P2X7R in myocytes would be therapeutically beneficial target for treating the PA-induced cells or HFD-induced rats.

Mounting evidence suggests that regular exercise, a lifestyle treatment option, is beneficial for many diseases. For example, aerobic exercise attenuated obesity-induced inflammation in skeletal muscle and liver (Sun et al., 2004) and improved lung function in asthma by reducing inflammation (Jung et al., 2013). In the circulatory system, exercise reduced the heart rate (Freitas et al., 2017) and relieved cardiac oxidative stress through inhibiting LOX-1 receptor expression (Machado et al., 2017). In addition, exercise-based cardiac rehabilitation could reduce hospitalization rates, cardiovascular mortality, and improve quality of life (Riahi et al., 2015). What’s more, in our study, we found that regular aerobic exercise effectively prevented body weight gain, alleviated lipid metabolism in HFD treated rats. In addition, the serum IL-1β level, caspase-1 and NLRP3 gradually decreased in all the rats that were subjected to exercise treatments. Similarly, exercise decreased the hallmark of apoptosis and fibrosis in cardiac tissue of HFD rats such as expression of caspase-3, Bax, and collagen I deposition. These results suggested that regular aerobic exercise effectively improved cardiac remodeling. Several mechanisms might be involved in this process during rats with HFD, such as chronic inflammatory processes, mainly induced by the proinflammatory cytokines NLRP3 and IL-1β, which subsequently contribute to the remodeling of heart tissues induced by HFD. A proinflammatory profile increased cells apoptosis and led to cardiac fibrosis. Remarkably, P2X7R expression in cardiac tissues was induced by HFD, reversed after exercise training. Moreover, several studies indicate that P2X7R expression is strongly associated with inflammasome via regulating the level of NLRP3 and IL-1β. To be more specific, the inhibition of P2X7R was linked to the reduction of apoptosis and fibrosis in myocytes treated with PA. These data indicated P2X7R may be as an intervening inflammatory gene by exercising training in improvements in heart remodeling in HFD rats.

**FIGURE 6:**
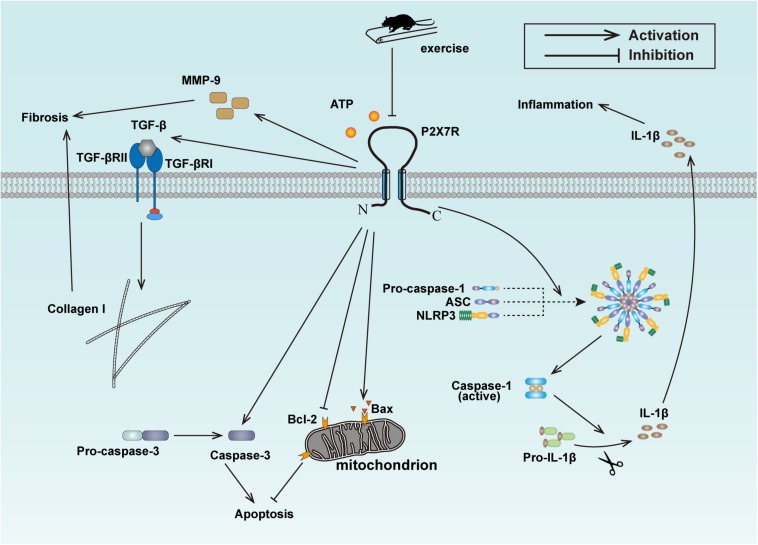
Schematic model for the exercise-mediated amelioration of inflammation, fibrosis, and apoptosis.

Given that exercise training reduced the expression of P2X7R, inhibited inflammation, fibrosis and apoptosis in cardiac tissues of HFD-induced rats, and that P2X7R inhibition also reversed the hallmark of fibrosis and apoptosis, alleviated inflammation in PA-treated cardiomyocytes, we concluded that aerobic exercise ameliorated cardiac remodeling of HFD rats at least in part via inhibiting P2X7R expression (Figure 6).

### Limitations

This study had a several limitations that needs to be addressed. (1) The results require further validation of P2X7*R^–/–^* mice. (2) Cardiac function data can help us further understand the protective effects of exercise and our study lacks echocardiographic assessment data in rats. (3) Find a more appropriate model to illustrate how exercise works at the cellular level. These issues will be solved in our future studies.

## Author Contributions

XC and HL conceived the study and drafted the original manuscript. KW contributed to the methodology. XL contributed to the data processing. WW and XH contributed to the statistics. ZH and YW designed the experiment and reviewed the manuscript.

## Conflict of Interest

The authors declare that the research was conducted in the absence of any commercial or financial relationships that could be construed as a potential conflict of interest.

## Supplementary Material

FIGURE S1 Schematic model of the experimental design.
