# Supplementary figures and images for: Aerobic Exercise Ameliorates Myocardial Inflammation, Fibrosis and Apoptosis in High-Fat-Diet Rats by Inhibiting P2X7 Purinergic Receptors
Source: Front Physiol. 2019 Oct 11;10:1286. doi: 10.3389/fphys.2019.01286 (PMC6798156; doi:10.3389/fphys.2019.01286)

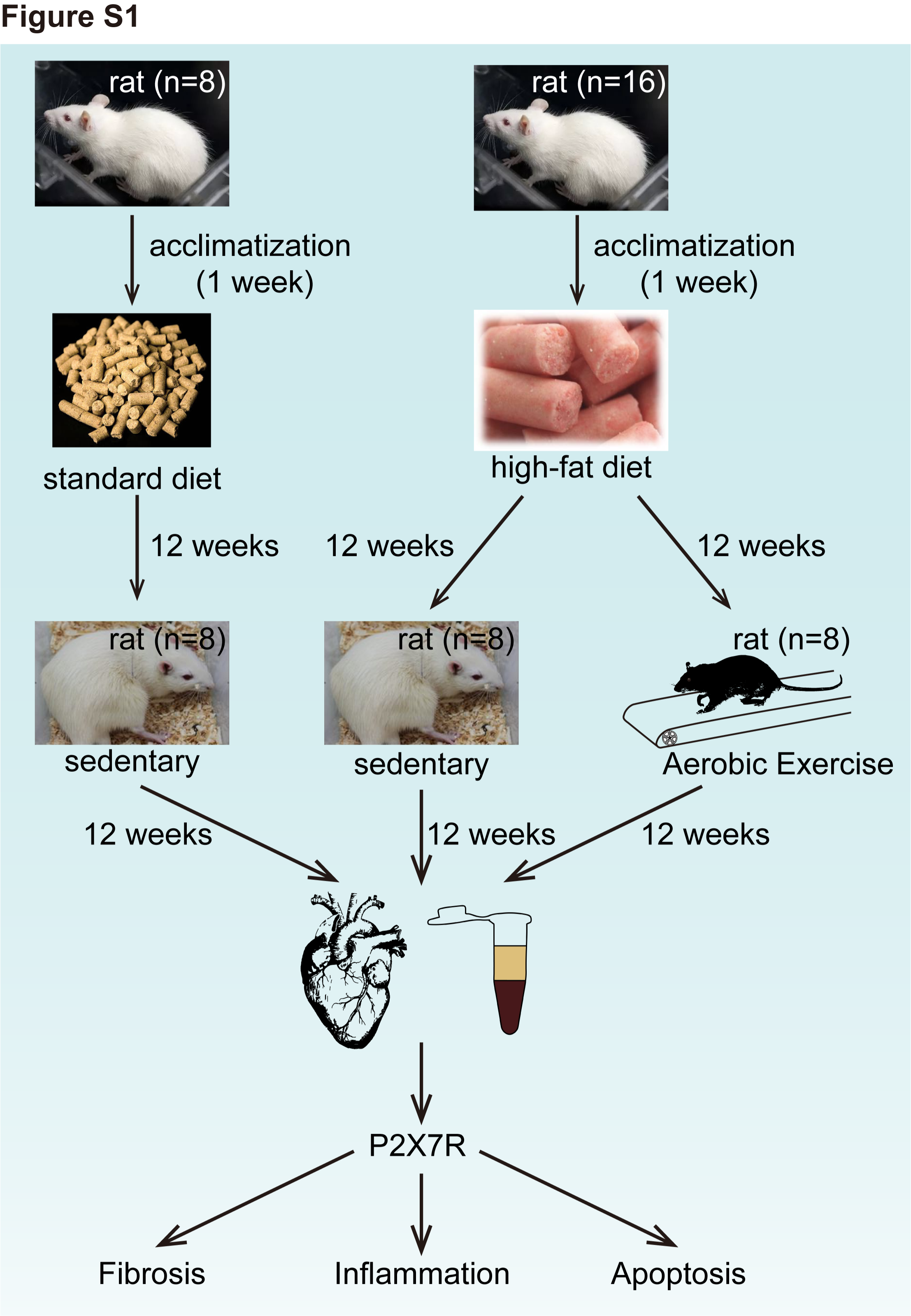

Supplement: FIGURE S1 — Schematic model of the experimental design. [file Image_1.TIF]
